# A study to improve the quality of writing clinic letters to patients attending the outpatient clinic

**DOI:** 10.1192/bjo.2021.565

**Published:** 2021-06-18

**Authors:** Irangani Mudiyanselage, Madhvi Belgamwar

**Affiliations:** 1 Derbyshire Health Care NHS Foundation Trust; 2 Consultant Psychiatrist, Derbyshire Healthcare NHS Foundation Trust

## Abstract

**Aims:**

In many countries (including the UK and Australia) it is still common practice for hospital doctors to write letters to patients’ general practitioners (GPs) following outpatient consultations, and for patients to receive copies of these letters. However, experience suggests that hospital doctors who have changed their practice to include writing letters directly to patients have more patient centred consultations and experience smoother handovers with other members of their multidisciplinary teams. (Rayner et al, BMJ 2020)

The aim of the study was to obtain patient's views to improve the quality of clinical letters sent to them, hence the level of communication and standards of care.

**Method:**

An anonymous questionnaire was designed and posted to collect information from patients attending one of the South County Mental Health outpatient clinic in Derbyshire. 50 random patients were selected between March to November 2020. Patients were asked to provide suggestions to improve the quality of their clinic letters written directly to them and copies sent to their GPs.

**Result:**

Out of 50 patients 48% (n = 24) responded. Majority of patients (92%) expressed their wish to receive their clinic letters written directly to them and 79% preferred to be addressed as a second person in the letters. More than half (54%, N = 13) of them would like to have letter by post. Majority of them (92%, N = 22) wished to have their letter within a week of their consultations.

Patients attending clinics felt that the communication could be better improved through writing clearly: a) reflection of what was discussed during the consultation b) updated diagnosis c) a clear follow-up plan d) current level of support e) medication change f) emergency contact numbers g) actions to be carried out by their GP and further referrals should there be any.

**Conclusion:**

Patients in community prefer to have their clinic letters directly addressing them in second person. It was noted that the letters needed to reflect accurately on what was discussed during the consultation in order to have patient centered consultations. This in turn would improve communication and thus rapport, trust and overall therapeutic relationship.

